# 11β-Hydroxysteroid dehydrogenases control access of 7β,27-dihydroxycholesterol to retinoid-related orphan receptor γ[S]

**DOI:** 10.1194/jlr.M092908

**Published:** 2019-07-04

**Authors:** Katharina R. Beck, Silvia G. Inderbinen, Sharavan Kanagaratnam, Denise V. Kratschmar, Anton M. Jetten, Hideaki Yamaguchi, Alex Odermatt

**Affiliations:** Division of Molecular and Systems Toxicology,* Department of Pharmaceutical Sciences, University of Basel, 4056 Basel, Switzerland; Immunity, Inflammation, and Disease Laboratory † National Institute of Environmental Health Sciences, National Institutes of Health, Research Triangle Park, NC 27709; Department of Applied Biological Chemistry§ Meijo University, Nagoya 468-8502, Japan

**Keywords:** oxysterol, 27-hydroxylase, mineralocorticoid receptor, retinoid-related orphan receptor gamma

## Abstract

Oxysterols previously were considered intermediates of bile acid and steroid hormone biosynthetic pathways. However, recent research has emphasized the roles of oxysterols in essential physiologic processes and in various diseases. Despite these discoveries, the metabolic pathways leading to the different oxysterols are still largely unknown and the biosynthetic origin of several oxysterols remains unidentified. Earlier studies demonstrated that the glucocorticoid metabolizing enzymes, 11β-hydroxysteroid dehydrogenase (11β-HSD) types 1 and 2, interconvert 7-ketocholesterol (7kC) and 7β-hydroxycholesterol (7βOHC). We examined the role of 11β-HSDs in the enzymatic control of the intracellular availability of 7β,27-dihydroxycholesterol (7β27OHC), a retinoid-related orphan receptor γ (RORγ) ligand. We used microsomal preparations of cells expressing recombinant 11β-HSD1 and 11β-HSD2 to assess whether 7β27OHC and 7-keto,27-hydroxycholesterol (7k27OHC) are substrates of these enzymes. Binding of 7β27OHC and 7k27OHC to 11β-HSDs was studied by molecular modeling. To our knowledge, the stereospecific oxoreduction of 7k27OHC to 7β27OHC by human 11β-HSD1 and the reverse oxidation reaction of 7β27OHC to 7k27OHC by human 11β-HSD2 were demonstrated for the first time. Apparent enzyme affinities of 11β-HSDs for these novel substrates were equal to or higher than those of the glucocorticoids. This is supported by the fact that 7k27OHC and 7β27OHC are potent inhibitors of the 11β-HSD1-dependent oxoreduction of cortisone and the 11β-HSD2-dependent oxidation of cortisol, respectively. Furthermore, molecular docking calculations explained stereospecific enzyme activities. Finally, using an inducible RORγ reporter system, we showed that 11β-HSD1 and 11β-HSD2 controlled RORγ activity. These findings revealed a novel glucocorticoid-independent prereceptor regulation mechanism by 11β-HSDs that warrants further investigation.

Oxysterols originate from cholesterol or cholesterol precursors and previously were mainly considered intermediates of bile acid and steroid hormone biosynthetic pathways. They display additional oxygen functionalities in the ring system and/or at the side chain of cholesterol, deriving through either enzymatic reactions and/or radical processes (1). Oxysterols are involved in several physiological processes including cholesterol, carbohydrate, and lipid homeostasis, immune system regulation, and neuronal development. Furthermore, recent research emphasized their role as bioactive lipids, contributing to the progression of various pathologies, such as neurodegenerative diseases, atherosclerosis, and cancer [reviewed in (2–6)]. Nevertheless, the metabolic pathways leading to the different oxysterols remain insufficiently understood and their biosynthetic origins need to be elucidated.

7-Ketocholesterol (7kC) is one of the most extensively studied oxysterols and particularly known for its pro-inflammatory and cytotoxic properties [reviewed in (1, 7, 8)], and it was recently reported to be associated with cardiovascular outcomes and total mortality in patients with coronary artery disease (9). However, it remains unclear whether 7kC itself or its metabolites caused these biological effects. In situ, 7kC is mainly generated by autoxidation of cholesterol during conditions of oxidative stress. In contrast to the low abundance of oxysterols in the circulation compared with cholesterol, 7kC is present at levels up to 10 μM in macrophage-derived foam cells in atherosclerotic lesions and in lenses of patients with cataract (10–12). In macrophages and in the retinal pigment epithelium, sterol 27-hydroxylase (CYP27A1) was found to convert 7kC to 7-keto,27-hydroxycholesterol (7k27OHC) by (13, 14). This metabolic step is ablated in macrophages derived from patients suffering from cerebrotendinous xanthomatosis, bearing a defect in the gene encoding CYP27A1 (13). These patients have normal circulating cholesterol, but increased 7kC levels, and are prone to the development of premature atherosclerosis (15).

Hepatic conversion of dietary 7kC to 7k27OHC is evident because the mitochondrial CYP27A1 is the first enzyme involved in the alternative biosynthesis pathway of bile acids in the liver (16, 17). However, a study with mice bearing a homozygous null mutation in the Cyp27 gene showed rapid and extensive hepatic 7kC metabolism, indicating the involvement of another enzyme (18). This enzyme was later proposed to be 11β-hydroxysteroid dehydrogenase type 1 (11β-HSD1), stereo-specifically converting 7kC into 7β-hydroxycholesterol (7βOHC) in humans, rats, and mice (19–21). Data from transgenic 11β-hsd1-deficient mice exhibiting an increased 7kC to 7βOHC ratio in liver tissue samples further supported these results (22).

The role of 11β-HSD1, well-known for the local conversion of glucocorticoids from inactive to active, in oxysterol metabolism and atherosclerotic plaque progression has been addressed by several studies [reviewed in (23)]. Interestingly, no direct accumulation of 7kC could be detected in the arterial wall of hsd11b1^−/−^ mice (24), and a loss of 11β-HSD1 function was rather associated with an atheroprotective and beneficial metabolic profile (25–27). Whether the observed favorable effects of diminished 11β-HSD1 activity are due to decreased intracellular levels of active glucocorticoids, or whether they are glucocorticoid-independent, remained unclear. This raised the hypothesis that 7kC acts as a precursor molecule requiring further downstream processing by another enzyme to exert its activity. Despite the extensive research focusing on the effects of 7kC and 7βOHC, only a weak interaction of 7kC with the arylhydrocarbon receptor and no cognate high-affinity receptors have been identified for 7kC and 7βOHC so far (28). However, for the sidechain-oxidized or the dihydroxylated oxysterols, such as 7β,27-dihydroxycholesterol (7β27OHC), several targets have been described [reviewed in (2)]. A recent study on Smoothened (Smo)-activating oxysterols suggested the conversion of 7βOHC to 7kC by 11β-HSD2, which required further metabolism by CYP27A1 to 7k27OHC in order to regulate Smo activity (29, 30). The enzymatic control of Smo by 11β-HSDs remains unclear, as both 7k27OHC and 7β27OHC were reported to promote Smo activity. Importantly, another receptor, retinoid-related orphan receptor γ (RORγ), was recently found to be activated by 7β27OHC, whereas 7k27OHC did not stimulate full-length RORγ activity in reporter gene assays (31). Nevertheless, a potential role for 11β-HSDs in the cell-specific metabolic availability of 7β27OHC and 7k27OHC, as they exert for glucocorticoids, has not yet been addressed. Thus, we investigated to determine whether 11β-HSDs are involved in the metabolism of 7-oxygenated 27OHCs and thereby could control the regulation of target receptors such as RORγ.

## MATERIALS AND METHODS

### Chemicals and reagents

7k27OHC, 7β27OHC, 7α27OHC, and 7β27OHC-d6 were obtained from Avanti Polar Lipids, Inc. (Alabaster, AL), [1,2-^3^H]cortisone, [1,2,6,7-^3^H]cortisol, and [1,2,6,7-^3^H] corticosterone from American Radiolabeled Chemicals (St. Louis, MO), and all other chemicals from Sigma-Aldrich (Buchs, Switzerland) of the highest grade available. Cell culture media were purchased from Sigma-Aldrich and Invitrogen (Carlsbad, CA) and FBS approved for use with the Tet-on system from Clontech (Mountain View, CA). UHPLC-grade purity methanol, acetonitrile, and formic acid were from Biosolve (Dieuze, France). 5H-1,2,4-triazolo(4,3-a)azepine,6,7,8,9-tetrahydro-3-tricyclo(3-3-1-13-7)dec-1-yl [T0504; also known as Merck-544 (25)] was purchased from Enamine (Kiev, Ukraine).

### Cell culture

HEK-293 cells and human SW-620 colon carcinoma cells were obtained from ATCC (Manassas, VA) and cultured in DMEM supplemented with 10% FBS, 4.5 g/l glucose, 100 U/ml penicillin/streptomycin, 2 mM l-glutamine, 10 mM HEPES (pH 7.4), and 10% MEM nonessential amino acid solution. Doxycycline-inducible RORγ stable Chinese hamster ovary (CHO) Tet-on cells were cultured in F12 medium containing 10% FBS approved for use with the Tet-on system and 100 U/ml penicillin/streptomycin (32, 33). Cells were regularly tested for the absence of mycoplasma.

### 11β-HSD-dependent metabolism of 7-oxygenated 27OHCs in intact cells

Fifty thousand stably transfected HEK-293 cells expressing either 11β-HSD2 [clone AT8 (34)] or 11β-HSD1 and hexose-6-phosphate dehydrogenase (H6PDH) [clone HHH7 (35)] were seeded in poly-l-lysine-coated 96-well plates and incubated for 24 h. H6PDH provides the cofactor NADPH and is required for an efficient oxoreduction reaction by 11β-HSD1 (36). The medium was changed to steroid- and phenol red-free DMEM (DMEM SF) for another 2 h and the cells were then treated with 1 μM of 7k27OHC in the presence or absence of 1 μM of T0504 (to inhibit 11β-HSD1) or 0.5 μM of 7β27OHC or 7α27OHC in the presence or absence of 5 μM of glycyrrhetinic acid (GA) (to inhibit 11β-HSD2). Metabolic conversion was determined after 0.5 h for 11β-HSD2 and after 1 h for 11β-HSD1.

SW-620 cells (500,000 cells/well) were seeded in 24-well plates and incubated for 24 h. The culture medium was replaced by DMEM SF containing 1 μM of 7βOHC in the presence or absence of 10 μM of GA for 3.5 h or 7 h.

Ice-cold acetonitrile:isopropanol (7:3, v/v) mixture containing either 100 nM deuterium-labeled 7β27OHC or corticosterone as internal standards were used to extract oxysterols from cell culture supernatants. Additionally, 1.5% (v/v) of a standard solution of the antioxidant butylated hydroxytoluene (BHT) and the peroxide reducing agent triphenylphosphine (TPP) (10 mg BHT and 25 mg TPP in 10 ml ethanol) was added (37). In experiments with SW-620 cells, oxysterols were extracted from cell supernatants and cells. The samples were incubated for 30 min at 4°C and 700 rotations per minute, centrifuged (16,000 *g*, 10 min, 4°C), and the supernatant evaporated to dryness under nitrogen to minimize oxidation from atmospheric oxygen. After reconstitution in 50 μl of methanol:ultra-pure water (1:1, v/v), the samples were again centrifuged (16,000 *g*, 10 min, 4°C) and the supernatants analyzed. Data (mean ± SD) were obtained from three independent experiments.

### 11β-HSD enzyme kinetic measurements

#### Preparation of microsomes.

Microsomes were prepared as described earlier with minor modifications (38). Briefly, HEK-293 cells stably expressing human 11β-HSD1 and H6PDH were cultured until they reached confluence. Then, they were washed with ice-cold PBS and resuspended to a final concentration of ∼2 × 10^7^ cells/ml in ice-cold buffer A [50 mM Tris (pH 7.5), 1 mM EDTA, 100 mM NaCl]. Typically, 6 ml of this suspension were prepared, corresponding to 1.2 × 10^8^ cells. Following homogenization with 20 strokes in a glass potter on ice, the preparation was sonicated and centrifuged (9.000 *g*, 4°C for 30 min). The supernatant was removed and subjected to ultracentrifugation at 105,000 *g*, 4°C for 70 min. The obtained pellet was resuspended in buffer B [20 mM Tris (pH 7.5), 1 mM EDTA, 10% glycerol] and the ultracentrifugation step repeated. The microsomal fraction was again resuspended in buffer B and the total protein concentration determined using BCA assay (39). The microsomes were immediately frozen on dry ice and stored at −80°C.

#### Determination of apparent K_M_ and V_max_ values.

Microsomes (1–20 μg) expressing 11β-HSD1 and H6PDH were incubated for 20 min with 1 mM of NADPH, 2 mM of glucose-6-phosphate, 0.1 units of glucose-6-phosphate dehydrogenase (from *Saccharomyces cerevisiae*), and different concentrations (62.5–2,000 nM) of 7k27OHC, adapted to a total assay volume of 50 μl with TS2 buffer [100 mM NaCl, 1 mM EGTA, 1 mM EDTA, 1 mM MgCl_2_, 250 mM sucrose, 20 mM Tris/HCl (pH 7.4)]. Oxysterols were extracted by mixing the samples with ice-cold ethyl acetate containing 100 nM deuterium-labeled 7β27OHC as internal standard and 1.5% (v/v) BHT/TPP solution as antioxidant. The samples were then incubated for 15 min at 4°C on a rotating mixer, shortly centrifuged, and the upper ethyl acetate phase transferred into a new tube. The extraction step was repeated and the ethyl acetate phases combined and evaporated to dryness under nitrogen. After reconstitution in 50 μl of methanol:ultra-pure water (1:1, v/v), the samples were centrifuged (4°C, 16,000 *g* for 10 min) and analyzed by UHPLC-MS/MS.

For 11β-HSD2 activity measurements, 0.8 μg of HEK-293 cell lysate stably expressing 11β-HSD2 was incubated with different concentrations (50–800 nM) of 7β27OHC and 500 μM of NAD^+^ for 10–30 min. Oxysterols were extracted as described in the 11β-HSD-dependent metabolism of 7-oxygenated 27OHCs in intact cells section.

The substrate conversion was kept below 30% in all reactions. Apparent kinetic parameters (K_M_ and V_max_) were determined by nonlinear regression and under the assumption of Michaelis-Menten kinetics. Data (mean ± SD) were obtained from three independent experiments.

### Sterol measurement by UHPLC-MS/MS

An Agilent 1290 Infinity UHPLC binary solvent delivery system including a temperature controlled autosampler and a column oven coupled to an Agilent 6490 triple quadrupole mass spectrometer with a jet stream electrospray ionization interface (AJS-ESI) (Agilent Technologies) was used to analyze oxysterols. A reversed-phase column (ACQUITY UPLC BEH C18, 1.7 μm, 2.1 × 150 mm; Waters, Wexford, Ireland) heated to 65 ± 0.8°C was applied for analyte separation. The flow-rate was 0.5 ml with solvent A [water/acetonitrile/formic acid (95/5/0.1, v/v/v)] and solvent B [water/acetonitrile/formic acid (5/95/0.1, v/v/v)]. The eluent gradient was set as follows: 0–4.5 min 45–97% B, 4.5–7 min 80% B (washout), and 7–9 min 45% B (column re-equilibration). Methanol/water (75/25, v/v) was used as needle and needle-seat flushing solvent for 10 s after sample aspiration. The samples were stored in the autosampler at 4°C until analysis.

Analyte fragmentation was directed in the positive ion mode for multiple reaction monitoring and source conditions defined by use of the integrated compound- and source-optimizer software module (Agilent Technologies; B.07.01). Multiple reaction monitoring transitions were defined as follows: 7k27OHC (*m/z* 417.3 → 417.3; retention time = 3.59 min), 7β27OHC (*m/z* 383.3 → 159.0; retention time = 3.45 min), 7α27OHC (*m/z* 383.3 → 383.3 and *m/z* 159.0; retention time = 3.57 min), 7β27OHC-d6 (*m/z* 407.38 → 159.1 and *m/z* 389.1; retention time = 3.42 min). Separation and retention time of 7k27OHC, 7α27OHC, and 7β27OHC are shown in supplemental Fig. S1. Source parameters were as follows: gas temperature 290°C, gas flow 14 l/min, sheath gas temperature 300°C, sheath gas flow 11 l/min, nozzle voltage 1,500 V, capillary voltage 3,000 V, cell accelerator voltage 4 V, fragmentation voltage 380 V, and nebulizer 20 psi.

For data acquisition and analysis, MassHunter Workstation Acquisition Software Version 07.01 SP1 and MassHunter Workstation Software Quantitative Analysis Version B.07.00/Build 7.0457.0, respectively (Agilent Technologies), were used.

### Inhibition of 11β-HSD-dependent glucocorticoid metabolism in cell lysates

11β-HSD1 and 11β-HSD2 enzyme activities were assessed in lysates of HEK-293 cells stably expressing the respective enzyme as reported earlier (40). Briefly, 11β-HSD1-dependent oxoreductase activity was determined by the incubation of lysates with 200 nM of radiolabeled cortisone, 500 μM of NADPH, and the test substance or vehicle for 10 min at 37°C. 11β-HSD2-dependent oxidation of cortisol was measured instead by the addition of 50 nM of radiolabeled cortisol and 500 μM of NAD^+^. The enzymatic reaction was stopped by introducing an excess amount of unlabeled cortisone and cortisol (1:1, 2 mM unlabeled each, in methanol). Cortisone and cortisol were separated using TLC and a chloroform and methanol (9:1, v/v) mixture. The bands were detected under UV light, scraped off the plate, and metabolism of the radiolabeled substrate was then evaluated by scintillation counting and the substrate conversion determined and compared with the control sample. Data (mean ± SD) were normalized to the control (DMSO) sample and obtained from at least three independent experiments.

### 11β-hsd2 activity in mouse kidney homogenates

The determination of 11β-hsd2 activity in mouse kidney homogenates was conducted as described earlier (41). Briefly, for homogenate preparation, kidneys from adult male mice (C57BL6) were snap-frozen and sonicated in homogenization buffer [250 mM sucrose, 10 mM HEPES (pH 7.4); 900 μl for 100 mg of tissue]. Then, the homogenate was centrifuged (2,000 *g*, 4°C for 10 min) and the total protein concentration determined. The enzymatic reaction was assessed using 0.075 mg/ml total protein of the kidney homogenate in incubation buffer [300 mM NaCl, 20 mM Tris/HCl, 1 mM EDTA, 10% glycerol (pH 7.7)], different concentrations of oxysterols, 50 nM of corticosterone (containing 50 nCi [1,2,6,7-^3^H] corticosterone), and 500 μM NAD^+^ at 37°C for 20 min. Corticosterone and 11-dehydrocorticosterone were separated using TLC. Substrate conversion was determined by scintillation counting. Data (mean ± SD) were normalized to vehicle control (DMSO) and obtained from at least three independent experiments.

### Mineralocorticoid receptor reporter gene assay

HEK-293 cells stably expressing 11β-HSD2 (AT8 cells) were seeded (100,000 cells per well) in poly-l-lysine-coated 24-well plates and incubated for 24 h. Then, the cells were transiently transfected with the mineralocorticoid receptor (MR) (300 ng/well), a TAT3-TATA luciferase reporter (450 ng/well), and a pCMV-lacZ β-galactosidase transfection control (25 ng/well) using the calcium phosphate precipitation method. At 6 h post-transfection, the cells were washed with DMEM and incubated for another 18 h before replacing the medium with cDMEM. The cells were cultured for another 2 h at 37°C. The medium was then again replaced with fresh cDMEM containing 50 nM cortisol in the presence or absence of different concentrations (78–2,500 nM) of 7k27OHC and 7β27OHC, and incubated for 24 h. The cells were lysed with Tropix lysis solution (Applied Biosystems, Foster City, CA) containing 0.5 mM of dithiothreitol and frozen at −80°C for at least 20 min. Luciferase activity was measured by the addition of D-luciferin-firefly substrate solution [0.47 mM D-luciferin, 53 mM ATP, 0.27 mM CoA, 0.13 mM EDTA, 33.3 mM dithiothreitol, 8 mM MgSO_4_, 20 mM tricine (pH 7.8)]. β-Galactosidase activity was determined using the Tropix kit. Data (mean ± SD) were normalized to the control (50 nM cortisol) and obtained from three independent experiments.

### RORγ reporter gene assays

Doxycycline-inducible RORγ CHO Tet-on cells were transfected with 11β-HSD1/H6PDH, 11β-HSD2. or pcDNA3 using the electroporation method according to the manufacturer’s protocol (Neon™ transfection system; Invitrogen). Briefly, the cells were resus per well). Three electric pulses of 1,620 V and 10 ms were delivered to the cells using a 100 μl gold tip. Then, the cells (20,000 cells per well) were seeded in 96-well plates using culture medium without antibiotics and incubated for 16 h. The medium was replaced with medium containing charcoal-dextran stripped FBS for 1.5 h before treating the cells with different concentrations of oxysterols. After 6 h, 1.25 μM of the inverse agonist SR2211 was added to the treatment for another 2 h. To induce ROR expression, the cells were additionally subjected to 1 μM doxycycline for a further 16 h. The cells were lysed in 20 μl Tropix lysis solution supplemented with 0.5 mM dithiothreitol. RORγ-mediated activation of the luciferase reporter was measured in 10 μl lysate by adding 100 μl of D-luciferin-firefly substrate solution. Data (mean ± SD) were obtained from at least three independent experiments.

### Molecular docking calculations

GOLD software (version 5.2, Cambridge Crystallographic Data Centre, Cambridge, UK) was applied to identify accurate docking poses for oxysterols in the binding sites of 11β-HSDs (42). Interactions found between the ligands and the proteins were further assessed by the use of LigandScout 3.12 (Inte:Ligand GmbH, Vienna, Austria). This software analyzes the interaction patterns between the protein and the docked ligand, based on the geometric distances, angles, and chemical functionalities (43).

For 11β-HSD1 docking calculations, the crystal structure with the Protein Data Bank entry 2BEL (https://www.rcsb.org/structure/2BEL) was selected for the human protein and for the mouse ortholog the Protein Data Bank entry 1Y5R (https://www.rcsb.org/structure/1Y5R). The co-crystallized ligands, carbenoxolone (2BEL) and corticosterone (1Y5R), were extracted from the ligand binding sites in order to be redocked and examined to determine whether GOLD could restore the original binding orientation. The binding site was defined as spherical space (9 Å, respectively 10 Å radius) centered on the hydroxyl group of Tyr183 in 2BEL (X = 3.08; Y = 19.19; Z = 13.65) and 1Y5R (X = 76.88; Y = 49.68; Z = 38.08). The applied settings were validated and showed RMSD values of 0.563 for carbenoxolone and 0.683 for corticosterone.

Two homology models were used for docking calculations for human (44) and murine (45) 11β-HSD2. The docking settings for the human 11β-HSD2 binding site were focused on following coordinates X = −20.5; Y = −0.60; Z = 11.38 surrounded by a 12 Å sphere. The residues, Arg212 and Ser152 (Ser219 in full-length protein), were edited as flexible. The settings for the mouse 11β-hsd2 binding site were centered on the coordinates X = −19.7; Y = 1; Z = 10.5 and defined as spherical space with a 10 Å radius. The amino acid, Trp276, was set flexible.

## RESULTS

### 11β-HSD1-dependent oxoreduction of 7k27OHC

**Fig. 1.**
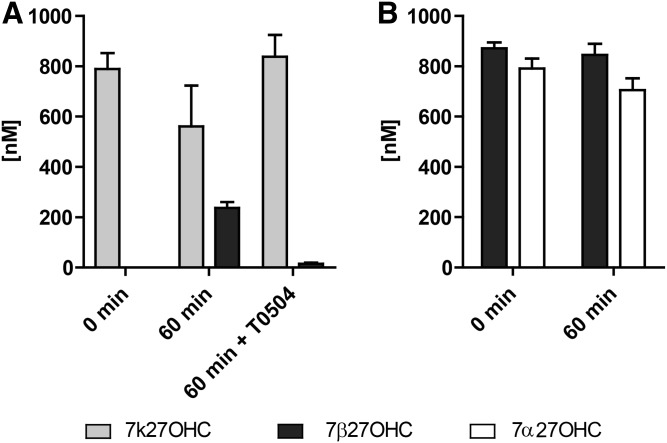
11β-HSD1-dependent metabolism of 7k27OHC in intact cells. HEK-293 cells stably expressing human 11β-HSD1 and H6PDH were treated with 1 μM of 7k27OHC with or without the 11β-HSD1 inhibitor T0504 (A) or 1 μM of 7β27OHC or 7α27OHC (B) for 60 min. 7k27OHC, 7β27OHC, and 7α27OHC concentrations were determined using UHPLC-MS/MS. Data represent the mean ± SD from three independent experiments.

In order to investigate whether 11β-HSD1 metabolizes 7k27OHC, HEK-293 cells stably expressing human 11β-HSD1 and H6PDH were treated with 1 μM of 7k27OHC in the presence or absence of the specific 11β-HSD1 inhibitor, T0504. 7k27OHC was stereo-specifically converted to 7β27OHC (**Fig. 1A**, supplemental Fig. S2), with an apparent enzyme velocity (V_app_) value of 0.52 ± 0.10 nmol·h^−1^·mg total protein^−1^ compared with a V_app_ of 1.59 ± 0.25 nmol·h^−1^·mg total protein^−1^ for cortisone (46) as substrate (**Table 1**). No 7α27OHC was generated. Moreover, neither 7α27OHC nor 7β27OHC was oxidized by 11β-HSD1 under the conditions applied and no isomerase activity could be detected (Fig. 1B). Enzyme kinetic analysis using microsomal preparations revealed an estimated apparent K_Mapp_ value of 39 ± 12 nM and an apparent V_max app_ value of 4.3 ± 0.2 nmol·h^−1^·mg total protein^−1^ for the oxoreduction of 7k27OHC to 7β27OHC by 11β-HSD1 (Table 1). Additionally, mouse 11β-hsd1 also showed the exclusive conversion of 7k27OHC to 7β27OHC without the formation 7α27OHC (data not shown).

**TABLE 1.** Kinetic parameters

Enzyme	Reaction	K_Mapp_ (nM)	V_max app_ (nmol·h^−1^·mg total protein^−1^)	V_app cell intact_ (nmol·h^−1^·mg total protein^−1^)
11β-HSD1	7k27OHC → 7β27OHC	39 ± 12	4.3 ± 0.2	0.52 ± 0.10*^a^*
	Cortisone → cortisol	250–500 (19, 34)	—	1.59 ± 0.25 (46)
11β-HSD2	7β27OHC → 7k27OHC	56 ± 11	10 ± 0.4	0.6 ± 0.1*^b^*
	Cortisol → cortisone	200 (34)	—	1.01 ± 0.35 (46)

Comparison of the oxoreduction of 7k27OHC and cortisone by 11β-HSD1 and the oxidation of 7β27OHC and cortisol by 11β-HSD2. Data represent mean ± SD from three independent experiments.

a A substrate concentration of 1 μM was used for the reaction.

b A substrate concentration of 500 nM was used.

### 11β-HSD2-dependent oxidation of 7β27OHC

**Fig. 2.**
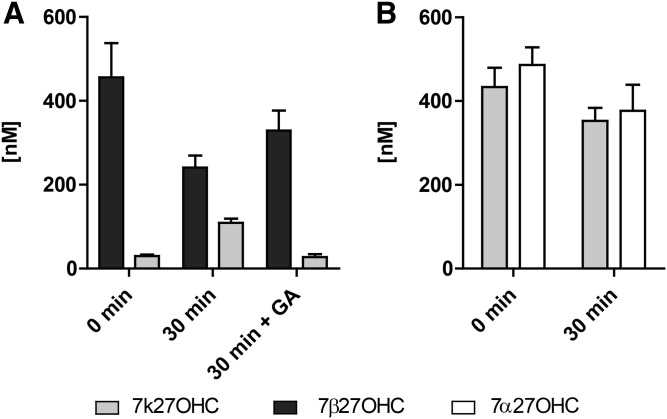
11β-HSD2-dependent metabolism of 7β27OHC in intact cells. HEK-293 cells stably expressing human 11β-HSD2 were treated with 500 nM of 7β27OHC with or without 5 μM of the 11β-HSD inhibitor GA (A) or 500 nM of 7k27OHC or 7α27OHC (B) for 30 min. 7k27OHC, 7β27OHC, and 7α27OHC concentrations were determined using UHPLC-MS/MS. Data represent the mean ± SD from three independent experiments.

The role of 11β-HSD2 in the metabolism of 7-oxygenated 27OHCs was then assessed in HEK-293 cells stably expressing the recombinant human enzyme. Treatment for 30 min with 500 nM of 7β27OHC or 7α27OHC showed the exclusive oxidation of 7β27OHC to 7k27OHC (**Fig. 2A**) and a lack of 7α27OHC metabolism (Fig. 2B, supplemental Fig. S2). Apparent enzyme velocity values were determined as 0.6 ± 0.1 nmol·h^−1^·mg total protein^−1^ for 7β27OHC and 1.01 ± 0.35 nmol·h^−1^·mg total protein^−1^ for cortisol (46) (Table 1). Furthermore, neither an oxoreductase nor an isomerase activity of 11β-HSD2 could be detected (Fig. 2B). Kinetic analyses of the oxidation reaction of 7β27OHC to 7k27OHC revealed a K_Mapp_ value of 56 ± 11 nM and a V_max app_ of 10 ± 0.4 nmol·h^−1^·mg total protein^−1^, following a Michaelis-Menten-type response (Table 1).

### Metabolism of 7βOHC by CYP27A1 and 11β-HSD2

**Fig. 3.**
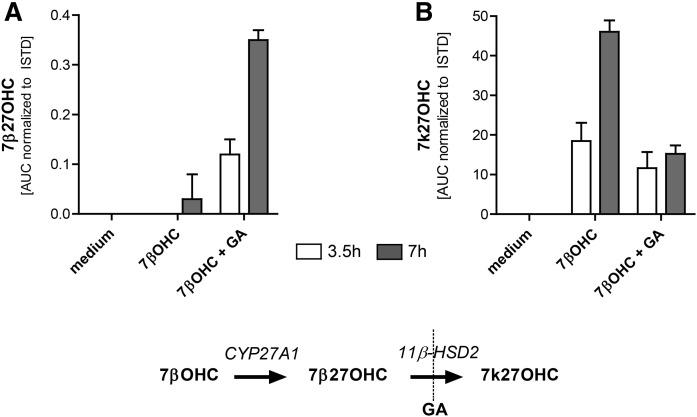
Metabolism of 7βOHC in SW-620 cells endogenously expressing CYP27A1 and 11β-HSD2. SW-620 cells were treated with 1 μM of 7βOHC in the presence or absence of 10 μM of GA for 3.5 h (white bars) or 7 h (gray bars). Qualitative analysis of 7β27OHC (A) and 7k27OHC (B) using the respective areas under the curve (AUC) normalized to the ISTD are illustrated. Data represent the mean ± SD from three independent experiments.

It was recently suggested that 7β27OHC formation includes in a first step an oxidation from 7βOHC to 7kC by 11β-HSD2, followed by hydroxylation at position 27 by CYP27A1 to 7k27OHC and then reduction of 7k27OHC to 7β27OHC by reactive oxygen species (29). Because reductive reactions by reactive oxygen species are highly unlikely, we tested the hypothesis of 27-hydroxylation of 7βOHC and subsequent oxidation to 7k27OHC in SW-620 colon cancer cells endogenously expressing CYP27A1 and 11β-HSD2. SW-620 cells were treated with 1 μM of 7βOHC in the presence or absence of the 11β-HSD2 inhibitor, GA, and the formation of 7β27OHC (**Fig. 3A**) and 7k27OHC (Fig. 3B) was evaluated. 7β27OHC was generated in a time-dependent manner from 7βOHC by CYP27A1 and then further oxidized by 11β-HSD2 to 7k27OHC.

### Oxysterol-dependent inhibition of 11β-HSD enzyme activities

**Fig. 4.**
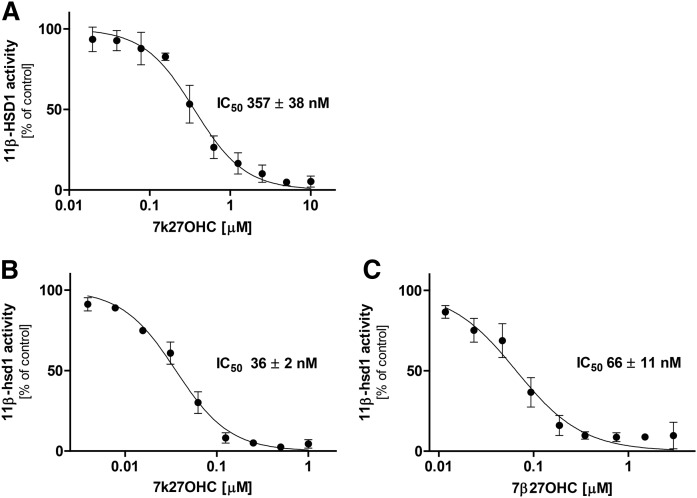
Inhibition of human (A) and murine (B, C) 11β-HSD1-dependent oxoreduction of cortisone by 7k27OHC and 7β27OHC. HEK-293 cell lysates expressing recombinant human 11β-HSD1 and H6PDH or murine 11β-hsd1 were incubated with 200 nM of radiolabeled cortisone, 500 μM of NADPH, and increasing concentrations of 7k27OHC (A, B) and 7β27OHC (C) for 10 min at 37°C. Cortisone to cortisol conversion was determined and compared with the enzyme activity in the control samples (0.1% DMSO). Data represent the mean ± SD from three independent experiments.

To assess the potential of 7-oxygenated 27OHCs to interfere with the metabolism of glucocorticoids, oxysterol-dependent inhibition of 11β-HSD enzyme activities were measured in lysates of HEK-293 cells expressing the respective enzymes. 7k27OHC inhibited the 11β-HSD1-dependent conversion of cortisone to cortisol with an IC_50_ value of 357 ± 38 nM for the human enzyme (**Fig. 4A**) and of 36 ± 2 nM for the mouse ortholog (Fig. 4B). Only weak inhibition of human 11β-HSD1 (IC_50_ value ≥3 μM) was detected for 7β27OHC, whereas 7α27OHC did not inhibit the enzyme activity at concentrations up to 10 μM (data not shown). However, mouse 11β-hsd1 enzyme activity was potently inhibited by 7β27OHC with an IC_50_ value of 66 ± 11 nM (Fig. 4C) and 1 μM 7α27OHC revealed ∼30% rest enzyme activity (data not shown).

**Fig. 5.**
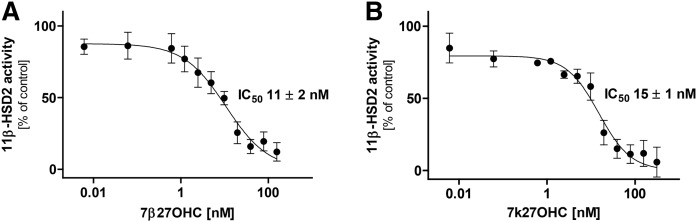
Inhibition of human 11β-HSD2-dependent oxidation of cortisol by 7β27OHC and 7k27OHC. Lysates of HEK-293 cells expressing recombinant human 11β-HSD2 were incubated with 50 nM of radiolabeled cortisol, 500 μM of NAD^+^, and increasing concentrations of 7β27OHC (A) and 7k27OHC (B) for 10 min at 37°C. Cortisol to cortisone conversion was determined and compared with the enzyme activity in the control samples (0.1% DMSO). Data represent the mean ± SD from three independent experiments.

11β-HSD2-dependent conversion of cortisol to cortisone was effectively inhibited by 7β27OHC and 7k27OHC with IC_50_ values of 11 ± 2 nM and 15 ± 1 nM, respectively (**Fig. 5**); however, 7α27OHC did not inhibit (not shown). Mouse kidney homogenates, expressing high levels of 11β-hsd2, were used to determine the interference of oxysterols with the metabolism of corticosterone to 11-dehydrocorticosterone (11-DHC). Neither 7β27OHC nor 7α27OHC inhibited the 11β-hsd2 activity, whereas 7k27OHC was a weak inhibitor with an IC_50_ value of 1.65 ± 0.4 μM (supplemental Fig. S3). Because diminished 11β-HSD2 activity has been associated with proinflammatory effects and an accelerated formation of atherosclerotic lesions in Apoe^−/−^11β-hsd2^−/−^ double-knockout mice, due to cortisol-dependent activation of the MR (47), 11β-HSD2-MR-dependent reporter gene assays were performed. For this purpose, HEK-293 cells stably expressing 11β-HSD2 were transfected with plasmids for MR and a MR-dependent luciferase reporter gene prior to treatment with cortisol and 7-oxygenated 27OHCs. Following inhibition of 11β-HSD2 by 7β27OHC and 7k27OHC, less cortisol was inactivated to cortisone and thereby an increased (∼3.5 times) cortisol-dependent MR activation (with EC_50_ values of 354 ± 55 nM for 7β27OHC and 331 ± 121 nM for 7k27OHC) compared with the exclusive activation by 50 nM cortisol could be detected (supplemental Fig. S4). Importantly, neither 7β27OHC nor 7k27OHC was able to stimulate MR activity by itself (data not shown).

### 11β-HSD docking calculations

**Fig. 6.**
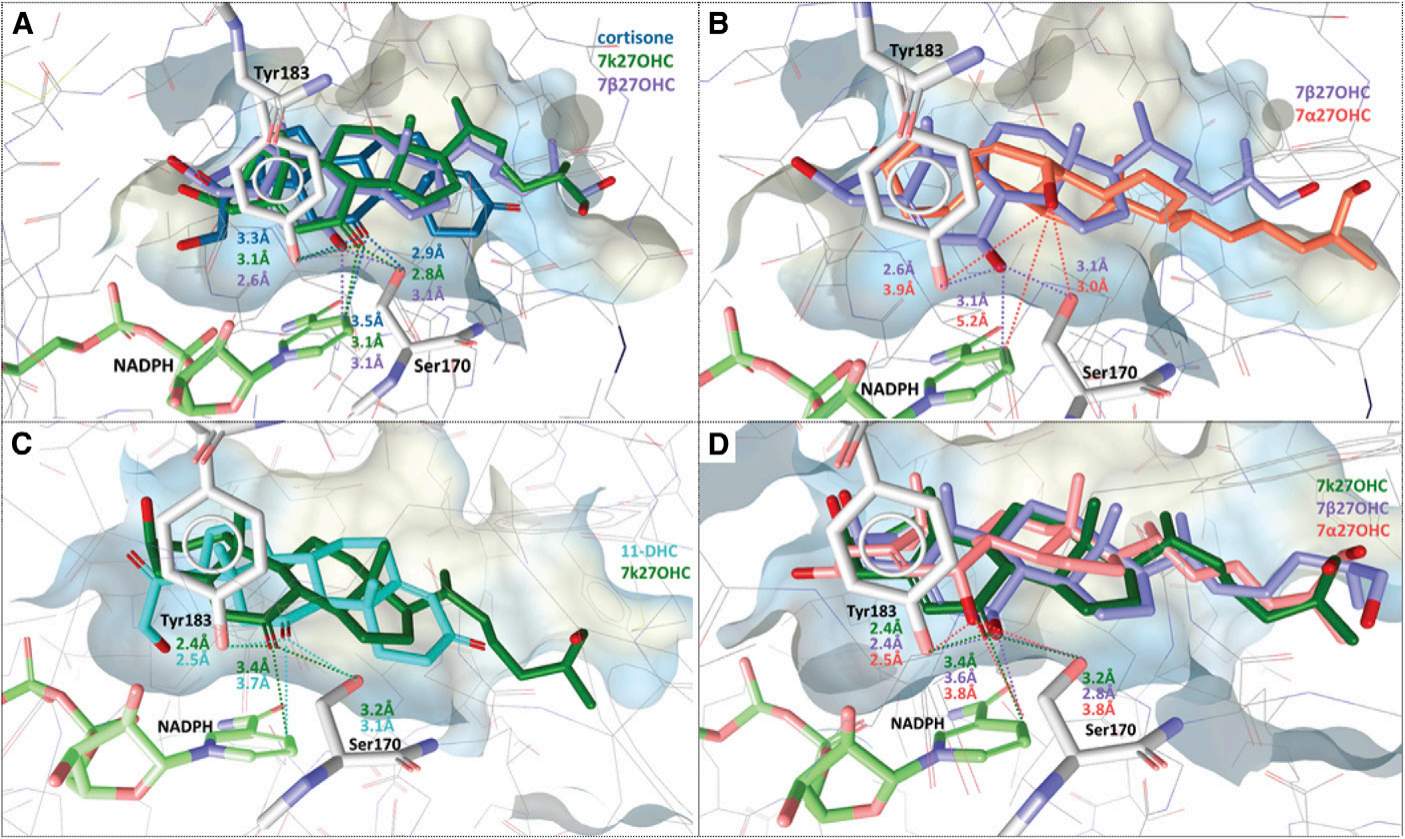
Predicted binding of cortisone, 11-DHC, 7k27OHC, 7β27OHC, and 7α27OHC to the binding pocket of human (A, B) and murine (C, D) 11β-HSD1. A: Representative binding poses of cortisone (blue), 7k27OHC (green), and 7β27OHC (purple). Important interactions for protein-ligand binding and the cofactor are shown in stick style and corresponding distances in ångstroms are indicated as dashed lines (same color code as docked ligands). B: Binding mode of 7β27OHC and 7α27OHC (salmon pink). C: Representative binding poses of 11-DHC (turquoise) and 7k27OHC. Important interactions for protein-ligand binding and the cofactor are shown in stick style and corresponding distances in ångstroms are indicated as dashed lines (same color code as docked ligands). D: Binding mode of 7k27OHC, 7β27OHC, and 7α27OHC.

Molecular docking calculations were implemented to predict the binding orientations of 7k27OHC, 7β27OHC and 7α27OHC in the substrate binding pockets of 11β-HSDs and, thus, providing a potential explanation for the results obtained from the enzyme activity measurements. 7k27OHC, cortisone, and 11-DHC were found to fit within comparable distances to the nicotinamide ring of the cofactor (NADPH) as well as to the catalytic residues Tyr183 and Ser170 of human and mouse 11β-HSD1 (**Fig. 6A**, C). Moreover, the enzymatic product, 7β27OHC, was orientated toward the important residues of the catalytic triad and the cofactor within similar distances as the substrates to 11β-HSD1 of both species (Fig. 6B, D). Compared with 7β27OHC, 7α27OHC adopted a slightly turned upwards binding orientation in the binding cavity of human 11β-HSD1 and, therefore, showed increased distances to Tyr183, Ser170, and particularly to NAPDH (Fig. 6B). However, docking analysis of 7α27OHC to mouse 11β-hsd1 displayed a more favorable alignment with similar distances as the substrates to the binding pocket (Fig. 6D).

**Fig. 7.**
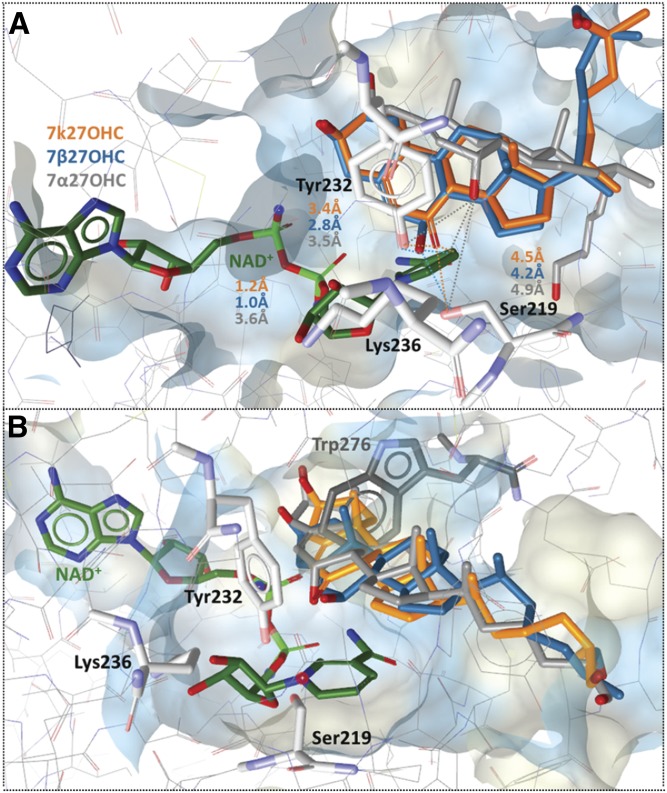
Predicted binding of 7k27OHC, 7β27OHC, and 7α27OHC to human (A) and murine (B) 11β-HSD2. Representative binding poses of 7k27OHC (orange), 7β27OHC (turquoise), and 7α27OHC (gray). Important interactions for protein-ligand binding and the cofactor are shown in stick style and corresponding distances in ångstroms are indicated as dashed lines (same color code as docked ligands).

Homology models were used for docking analyses due to the absence of 11β-HSD2 crystal structures. 7β27OHC and 7k27OHC were found in close distance to the cofactor NAD^+^ (1.0–1.2 Å) and the catalytic residue Tyr232 (2.8–3.4 Å), whereas 7α27OHC fitted again in a turned upwards position with enlarged distances into the substrate binding site of human 11β-HSD2 (**Fig. 7A**). A considerable species-specific difference was found between the murine and the human 11β-HSD2 substrate-binding cavity: in close distance to the catalytic residues and the cofactor-binding site, the residue Trp276 limited the size of the substrate-binding pocket and constricted the murine enzyme. In the human model, however, this residue was located outside of the binding cavity. Hence, steric clashes with Trp276 were observed when docking 7β27OHC, 7α27OHC, and 7k27OHC to mouse 11β-hsd2, providing a possible explanation for their weak or inactive interaction potential (Fig. 7B).

### Prereceptor regulation of RORγ by 11β-HSDs

**Fig. 8.**
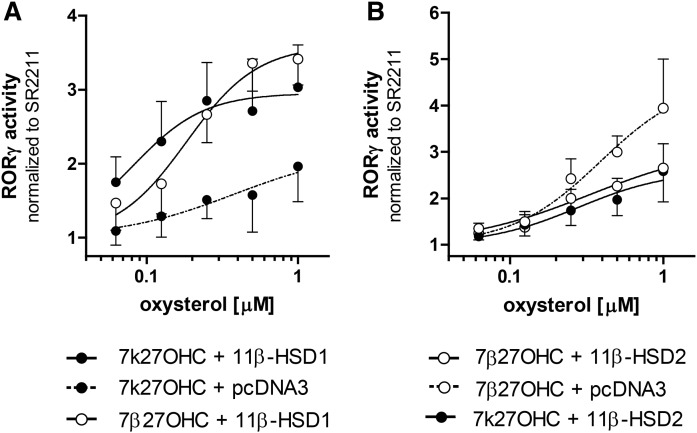
RORγ-mediated activation of the luciferase reporter by 7,27OHCs in CHO Tet-on cells transfected with either 11β-HSD1 (A) or 11β-HSD2 (B). Reversal of the inhibitory effect of the inverse agonist SR2211 by the addition of 7k27OHC or 7β27OHC and its dependence on 11β-HSD1 or 11β-HSD2. The reporter activation was illustrated as fold change over the inverse agonist SR2211. Data represent the mean ± SD from at least three independent experiments.

A recent study identified 7β27OHC as an endogenously occurring RORγ agonist, whereas 7k27OHC was not able to stimulate full-length RORγ activity in reporter gene assays (31). Thus, the capacity of 11β-HSDs to act as metabolic regulators, providing or inactivating the RORγ ligand, was examined. To obtain these insights, CHO cells stably expressing a Tet-on RORγ vector and a RORE-Luc reporter were additionally transfected with either an hH6PDH-IRES-hHSD11B1 construct (**Fig. 8A**) or 11β-HSD2 (Fig. 8B) and subjected to treatment with 7k27OHC and 7β27OHC. 7k27OHC was capable of reversing the inhibitory effect of the RORγ antagonist, SR2211, in the same manner as 7β27OHC (white dots, continuous line) upon coexpression of RORγ with 11β-HSD1 (black dots, continuous line) compared with the control (black dots, dashed line). Treating 11β-HSD2 expressing RORγ Tet-on cells with 7β27OHC could not restore RORγ activity and showed the same weak response as 7k27OHC. Additionally, treating the cells with 7α27OHC exhibited the same weak concentration-dependent effect as 7k27OHC when expressing only the control plasmid (data not shown).

## DISCUSSION

**Fig. 9.**
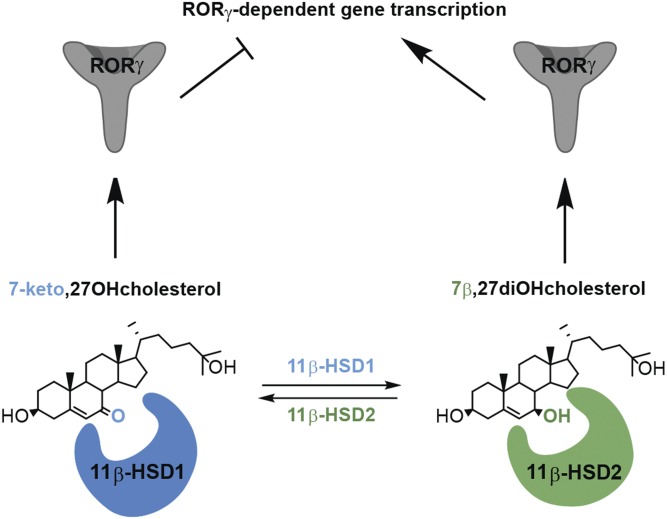
Control of RORγ ligand 7β27OHC availability by 11β-HSDs.

The family of oxysterols comprises a large number of compounds; however, their abundance in biological systems is low compared with cholesterol (1). Because several enzymes may act on a given metabolite and the enzymes involved often exhibit promiscuous substrate specificities, the analysis of their biosynthetic pathways is challenging. Moreover, oxysterols possess a high susceptibility for autoxidation, predisposing them to biased study results (1). For example, 7β27OHC can be formed either by oxoreduction of 7kC through 11β-HSD1 to 7βOHC and then further by hydroxylation to 7β27OHC through CYP27A1 or via direct hydroxylation of autoxidation-derived 7βOHC by CYP27A1. We showed two novel alternative biosynthetic pathways, i.e., the 11β-HSD1-dependent generation of 7β27OHC and the 11β-HSD2-dependent formation of 7k27OHC. Besides the 11β-HSD-dependent interconversion of the mono-hydroxylated oxysterols, 7kC and 7βOHC (19–21, 29), the only reported enzymatic reaction catalyzed by 11β-HSD2 involving oxysterols was, until now, the oxidation of cholestane-3β,5α,6β-triol to the oncometabolite, 6-oxo-cholestane-3β,5α-diol, and the reverse reduction reaction through 11β-HSD1 (48). Cholestane-3β,5α,6β-triol is formed from autoxidation-derived cholesterol-5,6-epoxide upon metabolism by cholesterol-epoxide hydrolase. The current study shows for the first time an involvement of 11β-HSDs in the interconversion of side chain-oxidized oxysterols, with relevance for RORγ activation (**Fig. 9**).

Kinetic analysis revealed remarkably high substrate affinities with estimated K_Mapp_ values in the lower nanomolar range of 11β-HSD1 and 11β-HSD2 for 7k27OHC and 7β27OHC, respectively. The affinity of 11β-HSD1 for 7k27OHC obtained in the present study is 6-25 times higher than the affinities previously reported for other endogenously occurring substrates (cortisone and 11-DHC, K_Mapp_ ∼250-500 nM (19, 34); 7kC, K_Mapp_ ∼500 nM (19); and 7oxoLCA, K_Mapp_ ∼1000 nM (49)). Moreover, the substrate affinity of 11β-HSD2 for 7β27OHC is ∼4 times higher than that of the previously reported cortisol (K_Mapp_ ∼200 nM) (34).

Apart from their own metabolic conversion by 11β-HSDs, 7k27OHC and 7β27OHC may alter other 11β-HSD-dependent reactions by competing with these substrates at the substrate-binding pocket and thereby inhibiting the corresponding reaction. In this regard, 7k27OHC moderately inhibited the oxoreduction of cortisone to cortisol by human 11β-HSD1 (IC_50_ value of 357 nM), therefore less likely being of physiological relevance. In contrast, the inhibitory capacities of 7k27OHC and 7β27OHC (IC_50_ values of 15 nM and 11 nM, respectively) to interfere with (human) 11β-HSD2-dependent oxidation of cortisol belong to the most potent endogenous 11β-HSD2 inhibitors discovered so far (50). Upon inhibition of 11β-HSD2, less cortisol is inactivated, resulting in higher cortisol availability and activation of the MR, as shown in transactivation assays. A role for endogenously formed compounds inhibiting 11β-HSD2 function was raised by the GA-like factor (GALF) hypothesis implicating an involvement in the etiology of not only essential hypertension but also colorectal cancer (50). Such GALFs, derived from peripheral tissues, the adrenal glands, or the gut microbiome could allow cortisol to act as a mineralocorticoid, leading to increased Na^+^ retention and hypertension (50, 51). Furthermore, they may cause elevated intracellular levels of active glucocorticoids, suppressing colorectal tumorigenesis and metastasis through different mechanisms.

An important question addresses the physiological relevance of plasma levels measured for potential GALF substances or oxysterols. Due to the lipophilic nature of oxysterols and the localized expression of oxysterol metabolizing enzymes, their formation and, thus, locally increased concentrations may not be represented by plasma concentrations. Hence, to understand oxysterol-related mechanisms, it is necessary to assess the tissue-specific or even cell-specific localization of biotransformation pathways and the involved substrate and product concentrations. The presented experiments using SW-620 colon carcinoma cells revealed a time-dependent formation of 7k27OHC by CYP27A1 and 11β-HSD2 from 7βOHC, with an accumulation of 7β27OHC upon inhibition of 11β-HSD2. Substantial concentrations of 7kC and 7βOHC may be reached in the gastrointestinal tract and in the liver following the intake of processed cholesterol-rich food. Both 7kC and 7βOHC were reported to induce inappropriate responses to the intestinal epithelium and thereby may disrupt the intestinal barrier integrity [reviewed in (52, 53)]. Whether these effects are due to 7-oxygenated 27OHC remains to be elucidated. Interestingly, innate lymphoid group 3 (ILC3) cells, which are expressed by the intestinal lymphoid tissue and characterized by the expression of the transcription factor, RORγt, have been described to contribute to the regulation of the intestinal barrier (53, 54). However, a hyperactivation of these RORγt-positive ILC3 cells has been described to contribute to intestinal inflammation (54). Because the nuclear receptors RORγ and RORγt are activated by 7β27OHC, but not or only weakly by 7k27OHC when full-length receptors are considered [(31), present study], 11β-HSDs may have an important role by either providing the active ligand 7β27OHC (cells expressing 11β-HSD1) or protecting the receptors from activation (cells expressing 11β-HSD2). Considering that an overstimulation of ILC3 cells could disturb the intestinal barrier function, a protective role for 11β-HSD2 by inactivating the RORγ ligand 7β27OHC to 7k27OHC is suggested, whereas 11β-HSD1 might aggravate the disturbance. Furthermore, recent evidence proposed that the intestinal lymphoid tissue plays a role in the pathology of inflammatory bowel disease (IBD) (55). A subgroup of ILC3s was shown to aggregate in ulcerative colitis and correlate with disease severity [reviewed in (53)]. Moreover, a dysregulation of colonic 11β-HSD expression has been associated with IBD progression and with increased 11β-HSD1 and decreased 11β-HSD2 expression (56–59). This would shift the balance form the inactive RORγ(t) ligand, 7k27OHC, to the agonist, 7β27OHC. However, whether this prereceptor regulation of RORγ and RORγt by 11β-HSDs is involved in the pathology of IBD requires further careful and thorough experimental investigation.

It needs to be noted that the mouse is of limited use to address the 11β-HSD-dependent regulation of RORγ(t). Our data indicate that while human and murine 11β-HSD1 both accept 7k27OHC as substrate and 7k27OHC effectively competes with cortisone at the substrate biding pocket, 7β27OHC is a high-affinity substrate for human 11β-HSD2 but seems to be unable to efficiently compete with cortisol for binding to the murine enzyme due to steric hindrance.

Nevertheless, the regulation of RORγ(t) by 11β-HSDs should be further investigated in relevant human cell models with endogenous expression of these proteins. However, in vitro assessment of the function of endogenously expressed RORγ using cell lines is highly complex and suitable cell models have not yet been identified. It is still unclear whether RORs are constitutively active receptors or whether their activities are driven by endogenously occurring (not yet characterized) ligands, including different oxysterols or intermediates of the cholesterol biosynthetic pathway. Compounds of the culture medium and of the cholesterol biosynthetic pathway in a cell endogenously expressing RORγ might control receptor activity and affect target gene expression. Furthermore, RORγ can be coexpressed with RORα and/or the transcriptional repressor, Rev-Erbα, that both can bind to the same RORE response element on promoter regions of target genes (60). The assessment of RORγ target gene expression can be strongly influenced by RORα and occasionally also by other nuclear receptors and receptor-associated coregulators that are tissue- and cell-specifically expressed. Therefore, the identification of a suitable cell model endogenously co-expressing RORγ with correspondingly responsive target genes and either 11β-HSD1 or 11β-HSD2 remains challenging. Alternatively, cells might be transfected with a RORγ-responsive reporter gene to achieve a more reliable readout.

To the best of our knowledge, very few human cell lines endogenously express substantial levels of 11β-HSD1, usually only following differentiation. A series of our own previous experiments with human and murine macrophages, myocytes, and adipocytes have shown that their differentiation is severely disturbed after transfection and that 11β-HSD1 expression is abolished, making these cell lines inapplicable to efficiently study RORγ regulation.

With respect to 11β-HSD2, we had used, in a series of preliminary experiments, SW-620 cells that express endogenous RORγ and also RORα and observed activation of a previously reported glucose-6-phosphatase luciferase construct (33). The potent RORγ inverse agonist, GSK2981278, was able to inhibit the reporter gene, although at high concentrations (IC_50_ of 10 μM); in contrast, the less potent inverse agonist, SR2211, failed to do so. Because endogenous expression of RORα and/or Rev-Erbα may interfere with RORγ activity, we then overexpressed RORγ in SW-620 cells, which resulted in a more pronounced response and a concentration-dependent decrease of the reporter gene activity by both GSK2981278 and SR2211 (with an IC_50_ of approximately 0.5 μM for the latter). Our preliminary data showed that inhibition of 11β-HSD2 in these cells by GA resulted in a trend toward enhanced RORγ activity (approximately 10% increase) compared with cells treated with 7β27OHC only. This suggests that the conversion of 7β27OHC to 7k27OHC by 11β-HSD2 partially protected RORγ from 7β27OHC but that the experimental setting was not suitable to detect 11β-HSD2-dependent protection of 7β27OHC-mediated RORγ activation. In addition, knockdown of 11β-HSD2 by siRNA instead of pharmacological inhibition did not result in a more pronounced effect. The following reasons might explain the failure to detect 11β-HSD2-dependent regulation of RORγ in our experimental setting: *a*) autoxidation (thereby inactivation of 7β27OHC and insufficient competition with the RORγ inverse agonist); *b*) further metabolism of the oxysterols during the experiment; *c*) generation of other RORγ agonists by the cell (for example, formation of 27OHC from cholesterol by CYP27A1); *d*) inappropriate timing (resulting in reporter gene activation before 7β27OHC is inactivated by 11β-HSD2); and *e*) insufficient competition with the inverse agonist (not reaching intracellular concentrations successfully competing with the inverse agonist). Thus, the SW-620 cell line has proven useful to study the enzymatic conversion of 7βOHC to 7k27OHC; however, it seems unsuitable to study the 11β-HSD2-mediated prereceptor regulation of RORγ.

Future efforts should focus on the identification of suitable cell models, including co-culture models to address possible paracrine effects, to investigate the control of RORγ activity by 11β-HSDs. Moreover, the role of 11β-HSDs on tissue-specific control of 7β27OHC levels should be studied in vivo in situations where the activities of these enzymes are impaired.

In conclusion, the present study demonstrated the stereospecific oxoreduction of 7k27OHC to 7β27OHC by 11β-HSD1 and the reverse oxidation reaction from 7β27OHC to 7k27OHC catalyzed by human 11β-HSD2 (Fig. 9). The apparent affinities of 11β-HSD1 and 11β-HSD2 for the 7-oxygenated oxysterols were equal or higher than for the glucocorticoids, indicating that they are preferred substrates. Furthermore, by supplying either 7β27OHC, the active ligand of RORγ, or inactivating 7β27OHC to 7k27OHC, a novel glucocorticoid-independent prereceptor regulation mechanism by 11β-HSDs could be shown. Future research to identify suitable cell models to characterize the control of RORγ activity by 11β-HSDs and to assess the relevance of 11β-HSDs in the metabolism of 7-oxygenated oxysterols is warranted.

## Supplementary Material

Supplemental Data
