# Supplementary material for: 11β-Hydroxysteroid dehydrogenases control access of 7β,27-dihydroxycholesterol to retinoid-related orphan receptor γ
Source: J Lipid Res. 2019 Jul 4;60(9):1535–46. doi: 10.1194/jlr.M092908 (PMC6718442; doi:10.1194/jlr.M092908)
Supplement: Supplemental Data [file supp_60_9_1535__index.html]

11β-hydroxysteroid dehydrogenases control access of 7β,27-dihydroxycholesterol to retinoid-related orphan receptor gamma — 11β-Hydroxysteroid dehydrogenases control access of 7β,27-dihydroxycholesterol to retinoid-related orphan receptor γ — Supplemental Data 

# 11β-Hydroxysteroid dehydrogenases control access of 7β,27-dihydroxycholesterol to retinoid-related orphan receptor γ

## Supplemental Data

- Supplemental Figure 1 (.pdf, 85 KB) - Extracted chromatograms of standards for 7k27OHC (black peak) and the stereoisomers 7β27OHC and 7α27OHC (blue peaks).
- Supplemental Figure S2 (.pdf, 226 KB) - Extracted sample chromatograms for 7k27OHC (black peaks) and 7β27OHC (blue peaks).
- Supplemental Figure S3 (.pdf, 69 KB) - 7k27OHC-dependent inhibition of mouse 11β-hsd2 enzyme activity.
- Supplemental Figure S4 (.pdf, 132 KB) - Cortisol-dependent MR transactivation in the presence of 11β-HSD2 and different concentrations of 7β27OHC (A) and 7k27OHC (B).
