# Supplementary material for: 11β-Hydroxysteroid dehydrogenases control access of 7β,27-dihydroxycholesterol to retinoid-related orphan receptor γ
Source: J Lipid Res. 2019 Jul 4;60(9):1535–46. doi: 10.1194/jlr.M092908 (PMC6718442; doi:10.1194/jlr.M092908)
Supplement: Supplemental Data [file 10.1194_M092908_jlr.M092908-1.pdf]

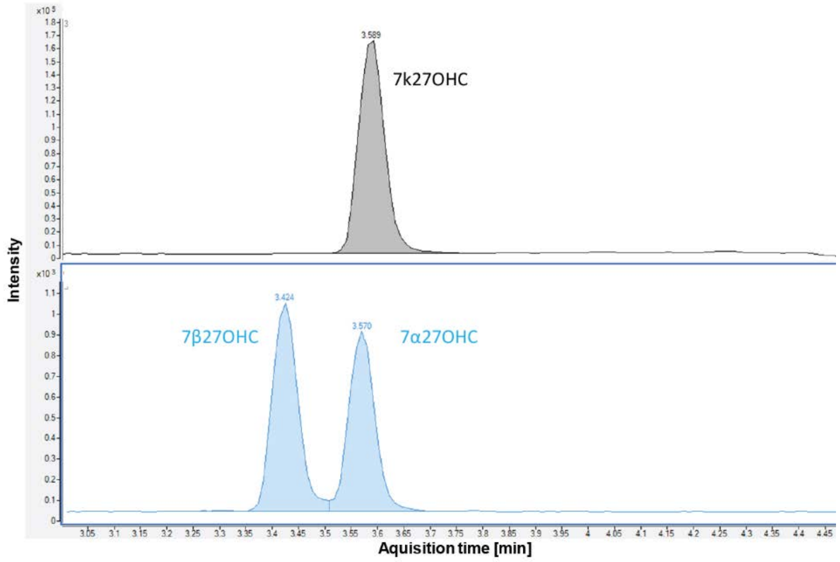

Supplemental Figure S1. Extracted chromatograms of standards for 7k27OHC (black peak) and the stereoisomers 7β27OHC and 7α27OHC (blue peaks).
