# Supplementary material for: 11β-Hydroxysteroid dehydrogenases control access of 7β,27-dihydroxycholesterol to retinoid-related orphan receptor γ
Source: J Lipid Res. 2019 Jul 4;60(9):1535–46. doi: 10.1194/jlr.M092908 (PMC6718442; doi:10.1194/jlr.M092908)
Supplement: Supplemental Data [file 10.1194_M092908_jlr.M092908-2.pdf]

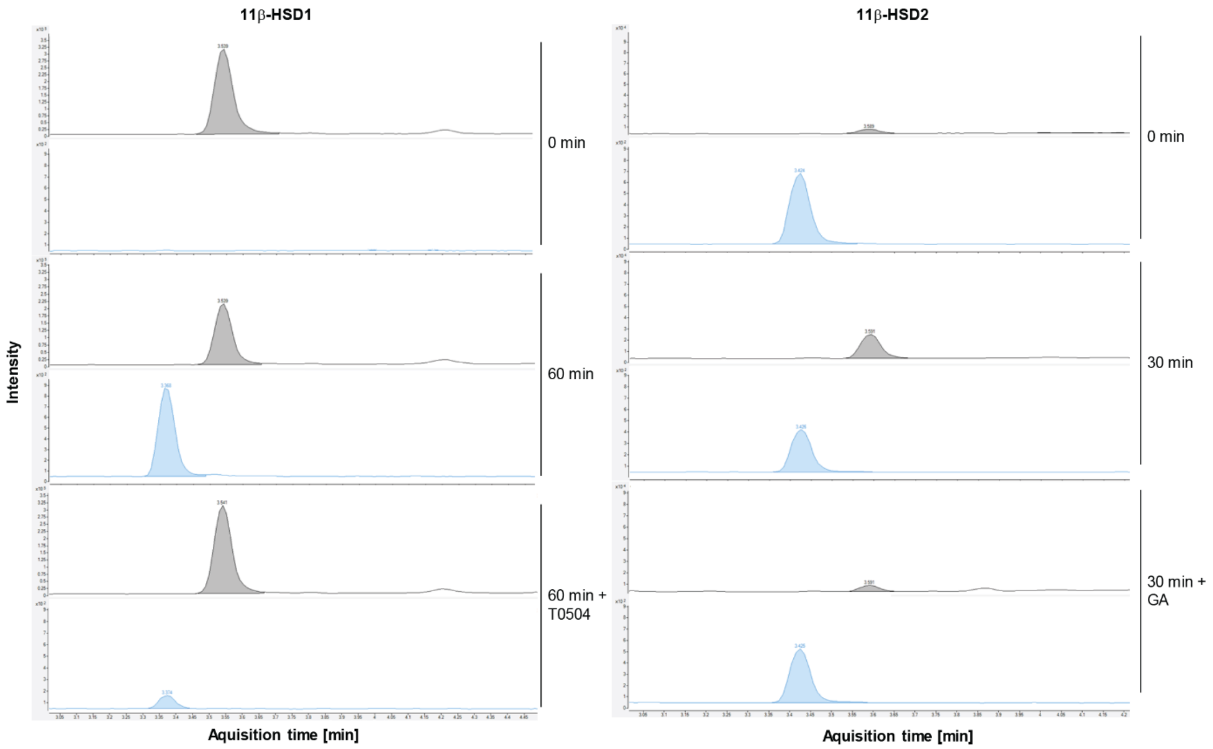

Supplemental Figure S2. Extracted sample chromatograms for 7k27OHC (black peaks) and 7β27OHC (blue peaks). The left panel displays the chromatograms for the 11β-HSD1-dependent oxoreduction of 7k27OHC to 7β27OHC in intact HEK293 cells at time point 0 min, 60 min, and 60 min in the presence of the specific 11β-HSD1 inhibitor T0504. The right panel shows the 11β-HSD2-dependent oxidation of 7β27OHC to 7k27OHC in intact HEK293 cells at 0 min and 30 min incubation time with or without the 11β-HSD2 inhibitor glycyrrhetinic acid (GA).
