# Supplementary material for: 11β-Hydroxysteroid dehydrogenases control access of 7β,27-dihydroxycholesterol to retinoid-related orphan receptor γ
Source: J Lipid Res. 2019 Jul 4;60(9):1535–46. doi: 10.1194/jlr.M092908 (PMC6718442; doi:10.1194/jlr.M092908)
Supplement: Supplemental Data [file 10.1194_M092908_jlr.M092908-3.pdf]

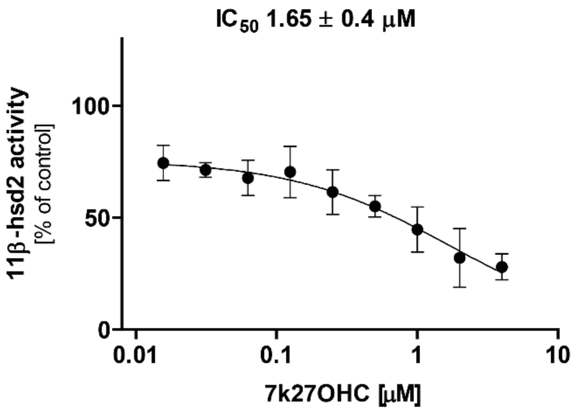

Supplemental Figure S3. 7k27OHC-dependent inhibition of mouse 11β-hsd2 enzyme activity. Mouse kidney homogenates were incubated with 50 nM radiolabeled corticosterone, 500 µM NAD^+ and increasing concentrations of 7k27OHC for 20 min at 37°C. The enzymatic conversion was determined and compared to the activity in the control samples (0.1% DMSO). Data represent mean ± SD from three independent experiments.
