# Supplementary material for: 11β-Hydroxysteroid dehydrogenases control access of 7β,27-dihydroxycholesterol to retinoid-related orphan receptor γ
Source: J Lipid Res. 2019 Jul 4;60(9):1535–46. doi: 10.1194/jlr.M092908 (PMC6718442; doi:10.1194/jlr.M092908)
Supplement: Supplemental Data [file 10.1194_M092908_jlr.M092908-4.pdf]

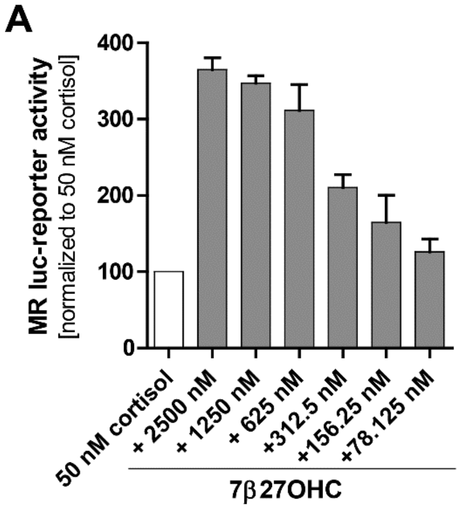

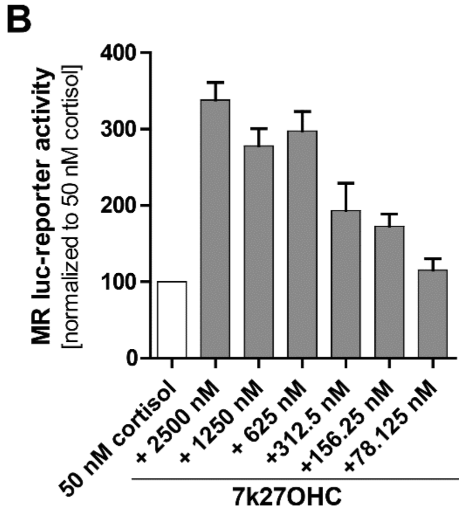

Supplemental Figure S4. Cortisol-dependent MR transactivation in the presence of 11β-HSD2 and different concentrations of 7β27OHC (A) and 7k27OHC (B). HEK-293 stably expressing 11β-HSD2 were transiently transfected with MR, a MR-sensitive luciferase reporter gene and a galactosidase transfection control. Data were normalized to 50 nM cortisol control and represent mean ± SD from three independent experiments.
